# Identifying *Sarcocystis* spp. infection in goats: a combined morphological, serological, and molecular approach

**DOI:** 10.1007/s00436-025-08519-8

**Published:** 2025-06-27

**Authors:** K. D. Steffen, M. L. Gos, E. Helman, J. M. Unzaga, R. O. Arias, G. Moré

**Affiliations:** 1 Immunoparasitology Laboratory, Faculty of Veterinary Science, La Plata National University, La Plata, 1900 Buenos Aires Argentina; 2 National Scientific and Technical Research Council (CONICET), Buenos Aires, 1900 Argentina; 3 Animal Production Department, Faculty of Agrarian and Forest Sciences, La Plata National University, La Plata, 1900 Buenos Aires Argentina; 4 Institute of Parasitology, Vetsuisse Faculty, University of Bern, Länggassstrasse 122, Bern, 3012 Switzerland

**Keywords:** Domestic goats, Kids, Sequencing and phylogeny, IFAT, MET, Argentina

## Abstract

Domestic goats (*Capra aegagrus hircus*) are intermediate hosts for three species of *Sarcocystis*: *S. capracanis*, *S. hircicanis*, and *S. moulei*. The present study aimed to describe and identify *Sarcocystis* spp. in goat/kid muscles utilizing optical and transmission electron microscopy, a serological method, and PCR-sequencing of individual cysts. Both *S. capracanis* and *S. hircicanis* were identified by light and transmission electron microscopy and molecular methods. No macroscopic cysts were detected in any of the examined animals (*n* = 90, from two Argentinean provinces); only microscopic sarcocysts of *Sarcocystis* spp. were detected in 17 of 19 adult goats (90%), but in no kids (*n* = 71) aged 37 to 290 days. Fourteen *18S rRNA* and the six *coxI* sequences from individual cysts highlighted high similarity (99.06–100% and 98.89–99.81%, respectively) with *S. capracanis* sequences from other regions. Two distinct groups of *S. capracanis 18S rRNA* sequences with high intra-group identities (99.42–100%) were identified, as well as another sequence that did not group. Each group was integrated from samples of both sampled provinces. Despite relatively extensive differences between these groups of sequences (up to 3.8%), the phylogenetic analyses positioned the three representative sequences all together in a branch with *S. capracanis* sequences but also containing *S. tenella*. In addition, one *18S rRNA* sequence showed a 100% identity with those of *S. hircicanis*. Two animals harbor *S. capracanis* and *S. hircicanis* cysts. Twelve out of 18 adult goats were seropositive by *Sarcocystis* spp. IFAT with titers ≤ 100. This is the first report that confirmed the infection and co-infection of *S. capracanis* and *S. hircicanis* in domestic goats from Argentina and evidenced a high variability in the *18S rRNA* fragment sequences of *S. capracanis*.

**Supplementary Information:**

The online version contains supplementary material available at 10.1007/s00436-025-08519-8.

## Introduction

Species of the genus *Sarcocystis* are intracellular parasites classified as cyst-forming coccidian protozoa within the *phylum* Apicomplexa (Dubey et al. 2016). Globally, sarcocystosis is one of the most prevalent parasitic infections in warm-blooded animals. Particularly in small ruminants, sarcocystosis is typically asymptomatic and infrequently associated with clinical signs or abortions (Buxton 1998). Domestic goats (*Capra aegagrus hircus*) are intermediate hosts (IH) for three species: *Sarcocystis capracanis* and *Sarcocystis hircicanis*, which utilize canids as definitive hosts (DH), and *Sarcocystis moulei* (syn. *S. hircifelis*), with felids as DH (Bittencourt et al. 2016; Dubey et al. 2016; Hu et al. 2016). The sarcocystosis prevalence in goats varies widely across countries, ranging from 13.3% to 100%, and depending on the diagnostic methodologies employed (Dubey et al. 2016; Hu et al. 2016). In South America, particularly in Brazil, Bittencourt et al. (2016) found *S. capracanis* sarcocysts in 91.6% of 120 carcasses of slaughtered goats. On the other hand, the *Sarcocystis* species that infect goats in Argentina are poorly studied.

Initial diagnosis often involves the detection of muscle cysts via optical microscopy; however, ultrastructural and molecular analyses are essential for a more accurate and comprehensive identification (Dubey et al. 2016). The PCR-sequencing of different markers, such as the small subunit ribosomal gene (*18S rRNA* gene), the large subunit ribosomal gene (*28S rRNA* gene), the mitochondrial cytochrome c oxidase subunit I gene (*coxI* gene), and internal transcribed spacer region 1 (*ITS1*), has been widely used (Moré et al. 2011; Kutty et al. 2015; Dubey et al. 2016). Some markers like the *28S rRNA* gene and *coxI* have proven more suitable to detect slight molecular differences between species that are phylogenetically related (Gjerde 2013; 2016; Hu et al. 2016). Serological techniques enable the detection of genus-specific antibodies in living animals (Dubey et al. 2016; Lindsay & Dubey 2020). Among the available methods, the immunofluorescence antibody test (IFAT) stands out for its high sensitivity (Dubey et al. 2016; Lindsay & Dubey 2020). Only one study has evaluated its use to detect *Sarcocystis* sp. infections in goats worldwide (Lefit et al. 1999).

Goat production in Argentina has a significant socioeconomic role, with a population of 4 million goats and approximately 46 thousand goat producers by 2024  (MAGyP, 2024). However, Argentina has no prevalence records, and the impact of sarcocystosis on goat production is unknown. This study aimed to describe and identify *Sarcocystis* spp. infection in goats and kids by utilizing optical and transmission electron microscopy, a serological method, and molecular characterization based on the *18S rRNA* and *coxI* markers.

## Materials and methods

### Farm and animals under study

The present study was conducted in three goat farms in Buenos Aires Province (E1, E2, and E3) and two in Salta Province (E4 and E5), Argentina. The E1 samples were collected consecutively over 3 years (2019, 2020, and 2021), resulting in a total of 69 samples from the Caprine Experimental Unit of the Faculty of Agricultural and Forestry Sciences, located in La Plata (− 34.911357, − 57.927385). Those from E2 (*n* = 6) and E3 (*n* = 4) were located in the district of General Paz, in the locality of Villanueva (− 35.64065, − 58.46460) and Ranchos (− 35.50756, − 58.35163) respectively. Samples from E4 (*n* = 7) were taken from *Cooperativa Agropecuaria* BRESEC in Seclantás (− 25.31257, − 66.24625) and from E5 (*n* = 4) in *Escuela Agropecuaria La Puerta* in Molino (− 25.24578, − 66.43572) (Supplementary Fig. 1). The production system in E1, E2, and E3 was essentially semi-intensive with goat milk and kids production, developed by controlling daytime grazing and penning with balanced supplementation. The production system in E4 and E5 was essentially extensive, with night enclosures without supplementation. The presence of dogs and cats was recorded in all farms.

### Samples

Skeletal muscle samples (masseters, base of tongue, and hamstrings) were collected from 90 goats (71 kids—37 to 290 days old—and 19 adult— ≥ 1 year-old goats) at slaughter or necropsy and transported refrigerated to the laboratory (LAINPA, FCV-UNLP). All *post-mortem* samples were collected by institutional ethical guidelines (protocol 97–1–19 T) and with the informed consent of farm owners. The collected samples by category are shown in Supplementary Table 1.

Additionally, blood samples (*n* = 18; *n*_Buenos Aires_ = 7 and *n*_Salta_ = 11) were obtained from adult goats during necropsy or slaughter by intracardiac or jugular puncture (3 to 5 ml), respectively. After centrifugation at 500 g, sera were stored in 1.5-ml microtubes at − 20 °C. These samples were analyzed for anti-*Sarcocystis* spp. antibodies by IFAT, using *Sarcocystis cruzi* bradyzoites (4 × 10⁶/ml) as heterologous antigens. Sera were diluted 1:50 in PBS and titrated to endpoint. An anti-goat IgG-FITC conjugate (Sigma Aldrich, USA) was used for detection followed by Moré et al. (2008) and Steffen et al. (2021; 2024).

### Direct microscopic examination, histopathology, and transmission electron microscopy (TEM)

Fresh skeletal muscles samples (50 g) from each animal (masseter, base of tongue, and hamstring) were macroscopically inspected at necropsy/slaughter for visible cysts and then processed for microscopic evaluation for *Sarcocystis* spp. infection. Tissues (5–10 g) were homogenized in 50 ml phosphate-buffered saline (pH 7.2), filtered, centrifuged, and the sediment was examined under an inverted microscope at 40 × magnification (Nikon, TMZ). Samples containing intact or portions of sarcocysts were considered positive. Sarcocysts were fixed in glutaraldehyde 2% for transmission electron microscopy (TEM). From all the positive samples, individual cysts or portions of them were preserved frozen at − 20 °C in 1.5 ml DNase-free microtubes for DNA extraction and molecular identification by polymerase chain reaction (PCR) and sequencing.

From five microscopy positive adult goats (from farms E1, E2, and E5), at least 20 cysts per sample were processed at the Central Service of Electron Microscopy (FCV-UNLP) and examined using a JEM 1200 EX II transmission electron microscope (JEOL, Japan). The ultrastructure of the cyst wall was photographed to identify characteristics and specific patterns of different species of *Sarcocystis* (Dubey et al. 2016).

Additionally, tongue and masseter muscle fragments from three adult goats with observed sarcocysts were fixed in 10% buffered formalin, processed by routine H&E staining, and examined histologically at the Pathological Anatomy Service (FCV-UNLP).

### PCR and sequencing

DNA extraction was performed from individual sarcocysts collected during the muscles direct microscopic examination, using a commercial kit according to the manufacturer’s instructions (PuriPrep T-kit, Inbio Highway, Argentina). A fragment of *Sarcocystis* spp., *18S rRNA* gene, was amplified by PCR using the primers *SarcoFext* and *SarcoRext* as previously described (Moré et al. 2013). Additionally, individual cysts that were positive in this PCR were processed by specific PCR for the fragments of the *cox*I gene using the forward primer *SF1* with two different reverse primers (*SR9* and *SRD8*) and the *ITS1* marker and flanking regions (*18S* and *5.8S* rRNA genes) with the forward and reverse primers *SU1F* and *5.8SR2*, respectively, according to protocols described by Gjerde (2013; 2016). Each PCR routine included a negative control (DNA extraction process control sample), a no-template control (NTC, ultrapure water), and a positive control (*S. falcatula*-like for *18S rRNA* and *ITS1* or *S. gigantea* DNA for *coxI*). Amplification products were purified using a commercial kit according to manufacturer instructions (Wizard SV clean-up system, Promega, USA and DNA Clean & Concentrator-5, Zymo Research, Irvine, USA) and submitted for Sanger sequencing to Macrogen Inc., South Korea, or to Microsynth, Balgach, Switzerland, with both primers used for each amplification. Sequences obtained were aligned and analyzed using Geneious Prime (2024.0.3 version). Consensus sequences obtained were compared with others reported in GenBank by BLAST analysis.

Representative *18S rRNA* and *coxI* sequences obtained from Argentinean goats were aligned with other sequences from *Sarcocystis* spp. retrieved from GenBank, using cervids and ruminants, among others, as IH. A phylogenetic tree was constructed using the neighbor-joining method based on the Tamura-Nei genetic distance model, 1000 bootstraps, and a *Toxoplasma gondii* sequence as the outgroup (Geneious Prime, 2024.0.3 version).

## Results and discussion

### Direct microscopic examination, histopathology, TEM, and IFAT

No macroscopic tissue cysts were detected in any of the examined animals (*n* = 90), and no microscopic cysts were found in the muscle homogenate of kids aged 38 to 290 days (*n* = 71). Adult goats tested positive for *Sarcocystis* spp. in 90% of cases (17/19; 95% CI: 65%–98%). The seventeen goats showed microscopic thick-walled cysts (less than 3 μm) compatible with *S. capracanis*, and two also had thin-walled cysts (less than 1 μm) compatible with *S. hircicanis*. The majority of observed cysts were thick-walled (≤ 3 μm) and showed cyst portions of 145–350 μm length and 35–95 µm width, with several complete cysts measuring up to 550 μm (*n* = 20), while a few thin-walled (≤ 1 μm) cyst portions were of 400–870 μm length and 40–55 µm width (*n* = 5) (Supplementary Fig.  2 A and 2B). Histopathology revealed the presence of fusiform basophilic intracellular bodies with septae, consistent with cysts of the genus *Sarcocystis* in the three goats analyzed (Supplementary Fig.  4 A). A low to moderate number of cysts, which appeared to be thick-walled and mature (Supplementary Fig. 4B), were identified in each sample. Notably, no inflammatory lesions were observed in the surrounding tissue.

The fact that sarcocysts were found in adult goats but not in kids suggests that infection occurs gradually over time through the ingestion of feed or water contaminated with *Sarcocystis* sporocysts (Dubey et al. 2016; Lindsay & Dubey 2020). Therefore, and according to our results, it seems that young animals, while still milking, are less exposed than subadults and adults (Dubey et al. 2016; Lindsay & Dubey 2020). The high *Sarcocystis* spp. cyst positivity rate observed in the adult animals in the present study (90%) is comparable to the rates documented in adult goats from Iraq (100%) (Zangana & Hussein 2017), Iran (99.4%) (Shekarforoush et al. 2005), and Egypt (79.4%) (Morsy et al. 2011). Other studies identified positivity rates ranging from 52.3 to 91.6% from Brazil, Iran, China, India, and Malaysia (Dafedar et al. 2008; Kutty et al. 2015; Bittencourt et al. 2016; Hu et al. 2016); however, the age of the sampled animals was not reported in these studies. These findings highlight the widespread occurrence of this parasitic infection in adult goats and suggest that, as more studies emerge, future research may explore regional variability influenced by climate, management, or diagnostics (Morsy et al. 2011; Dubey et al. 2016; Hu et al. 2016; Salehi et al. 2022). Macroscopic cysts were not detected in any animals from either province (0/90). Likewise, very low frequencies of *S. moulei* forming macroscopic cysts in goats have been reported in Iran and Egypt (Salehi et al. 2022; El-Morsey & Abdo 2024). The low prevalence of *S. moulei* may reflect limited goat–cat contact and environmental contamination with sporocysts in Argentina’s extensive farming systems, as previously suggested (Latif et al. 1999).

The morphology of *S. capracanis* and *S. hircicanis* differs in the ultrastructure of the cyst wall (Dubey et al. 2016; Hu et al. 2016). In the present study, 9/10 cysts collected and analyzed by TEM presented a cyst wall type compatible with *S. capracanis,* resembling the TEM wall type 14b (Dubey et al. 2016). This type displays upright finger-like or cylindrical protrusions. One cyst (from a sample also containing *S. capracanis* cyst) presented an ultrastructure compatible with *S. hircicanis*, conforms to the TEM wall type 7a (Dubey et al. 2016), with the cyst wall protrusions appeared hairy. Co-infection, by detecting both types of cysts in the same animal, was also described in goats in Japan (Saito et al. 1995), China (Hu et al. 2016), and Egypt (El-Morsey & Abdo 2024). These results showed the relevance of performing cyst identification by TEM as a confirmation method of the optical microscopy results to achieve proper species identifications (Dubey et al. 2016).

Additionally, twelve of the eighteen adult goats (5/7 from Buenos Aires and 7/11 from Salta) were seropositive for *Sarcocystis* spp. and showed low titers ≤ 100 by IFAT (Supplementary Fig. 5 and Table 2). Microscopic cysts were found in all seropositive goats (12/18). Of the 6 seronegative goats, 4 showed microscopic cysts. Serological evaluation using the IFAT technique revealed a 100% positive predictive value, and the negative predictive value was relatively low (33%). This phenomenon may be attributed to chronic infections in long-lived animals, where antibody titers decline over time due to immune tolerance or reduced antigenic stimulation (Dubey et al. 2016; Lindsay & Dubey 2020). In this study, three out of four seronegative goats with tissue cysts were over 5 years old, supporting the hypothesis that older animals, despite being infected, may exhibit diminished or undetectable antibody responses.

### PCR and sequencing

Screening *18S* PCR was positive in 31 individual cysts from 17 goats, and 19 amplicons were sequenced. Of the 19 samples, 16 were subjected to *coxI*-targeted PCR, and 6 were subjected to *ITS1*-targeted PCR, resulting in 6 and 4 readable sequences, respectively. The only primer combination producing acceptable *coxI* amplicons was SF1/SR9. Fourteen of the partial *18S rRNA* gene consensus sequences obtained (517–845 bp) showed an identity of 99.06–100% (100% coverage) with sequences reported as *S. capracanis* from Barbary sheep (*Ammotragus lervia*), Alpine ibex (*Capra ibex*), and domestic goat. One sequence (818 bp) resulted in 100% identity and coverage with a *S. hircicanis* sequence (KU820984). Four sequences could not yield consensus due to double peaks in chromatograms. A multiple alignment of the 14 *S. capracanis*-like obtained sequences from individual goat cysts revealed a relative homology of 99.42–100% among eight sequences (3S, 6S, 6003 K, MATC5/F, 2S, 8072D, 6003 C, 6003 F), and the closest matches corresponded to *S. capracanis* sequences, particularly MW832480 and MW832494 from Barbary sheep in Spain (99.81–100% identity), PQ963167-PQ963170 from Alpine ibex in Austria (99.83–100% identity), and PP657639 from an Egyptian goat (99.83% identity). Another five sequences (68/1C, 5004/1A, 61 F, MATC4/R, 5S) exhibited a homology of 99.49–100% among them but differed from the previously mentioned eight sequences by 15 to 17 nucleotide polymorphisms and 4 gaps (identity of 96.13–97.82%). These sequences exhibited high similarity to *S. capracanis* sequences, including MW832482, MW832485, MW832487, and MW832493 from Barbary sheep in Spain (99.51–99.88% identity) and also *S. tenella* sequences MW832470-MW832472 from the same host and KC209734 from a Norwegian sheep (98.82–99.49% identity). In addition, one sequence (4S) showed a relative homology of 98.45–99.15% and 97.62–98.34% with the eight and five sequences in each group, respectively. This sequence has a relatively lower identity (99.17–99.64%) to *S. capracanis* sequences from Barbary sheep in Spain (MW832480 and MW832494) and 99.06% with *S. capracanis* from Capra ibex from Austria (PQ963169). Each group of sequences was integrated from samples of both sampled provinces (Supplementary Table 2), suggesting independence and wide distribution of the observed sequence variants. Despite relatively extensive differences between these groups of sequences (up to 3.8%), the phylogenetic analyses positioned the three representative sequences from this study all together in a branch with *S. capracanis* sequences but also containing *S. tenella*, grouping the species producing thick-walled cysts in small ruminants and with canids as DH (Supplementary Fig. 6). A similar phylogenetic placement has been previously reported (El-Morsey & Abdo 2024). Nevertheless, the variability among all *S. capracanis-*like *18S* sequences from the present study resulted higher than the interspecific variability. The *18S* gene has been considered not discriminative enough among *Sarcocystis* spp. using small ruminants as IH (Gjerde 2013; Dubey et al. 2016; Delgado-de las Cuevas et al. 2021). Our results suggest that there are at least genotypes or variants within the morphologically identified as *S. capracanis*. Moreover, the extensive differences observed deserve further molecular studies using single cyst multilocus PCR and sequencing to confirm if we are dealing with recombinant genotypes or potential new species producing thick-wall cysts, possibly using canids as DH. A thin-walled cyst (61C, PQ288512) resulted in the highest similarity to *S. hircicanis* sequences, particularly KU820984 from a Chinese goat (100% identity) and OP430816 from an Egyptian goat (99.51% identity). This sequence positioned in the phylogenetic tree in a branch together with *S. hircicanis* from other regions and with *S. arieticanis*, which infects sheep and also employs canids as DH (Supplementary Fig. 6). While these species exhibit differences in intermediate host specificity, they are morphologically indistinguishable (Saito et al. 1995; Hu et al. 2016; Dubey et al. 2016; El-Morsey & Abdo 2024). Altogether, the phylogenetic assessment revealed a relatively close association between isolates of *S. capracanis* and *S. tenella*, and *S. hircicanis* with *S. arieticanis*, and also from a diverse range of intermediate hosts, including goats, sheep, Barbary sheep, and Alpine ibex. Other molecular studies have shown wild and domestic ruminants can harbor the same species and sometimes co-infections (Gjerde 2013; Dubey et al. 2016; Delgado-de las Cuevas et al. 2021). Therefore, the validity and separation of species should be discussed since there is no clear proof that they are interfertile and can recombine in the intestine of a canid DH.

Five *coxI* gene fragment sequences (1020–1038 bp) obtained showed a relative homology of 99.2–99.9% among them and 98.83–99.81% identity with previously described *S. capracanis* sequences from different hosts and regions (e.g., MW848331; MW848328). One of the samples (61F) showed a poor-quality *coxI* sequence; it was trimmed, resulting in a shorter consensus (542 bp) displaying an identity of 98.15–99.6% with the other five and 98.52–98.89% with *S. capracanis* sequences (MW848331; MW848328. Sequencing results, BLAST comparisons, and the number of sequences deposited in GenBank are shown in Supplementary Table 3. The six sequences obtained exhibited the closest similarity to MW848320, MW848328, and MW848331–MW848335 from Barbary sheep in Spain (98.52–99.81% identity) and to PQ998234 and PQ998236 from Alpine ibex in Austria (98.71–99.61% identity), KU820974 and KU820977 from goats in China (98.43–98.82% identity), and to PP668138 and PP668139 from goats in Egypt (98.81% identity). These results, together with the placement in the phylogenetic tree (Supplementary Fig. 7), confirmed that the mitochondrial gene *coxI* allowed a separation of what is considered *S. capracanis* from *S. tenella* (Dubey et al. 2016; Delgado-de las Cuevas et al. 2021). These findings reinforce the idea of a multilocus approach for species identification and delimitation within Sarcocystidae, improving our understanding of their biodiversity and evolution (Gjerde 2013; Dubey et al. 2016; Delgado-de las Cuevas et al. 2021). Unfortunately, the ITS1 amplification and sequencing failed in most of the attempts. Moreover, the only two short sequences obtained display low homology with other reported sequences. Possibly, the repetitive nature of this target could have impaired the performance of the PCR amplification and Sanger sequencing. More samples and using other primers targeting the ITS1 will be assayed to achieve proper sequences.

By a combination of microscopy, TEM, and molecular characterization, we found that two adult goats simultaneously contained *S. capracanis* and *S. hircicanis* cysts, indicating that both animals were co-infected.

So far, *S. capracanis* appears to be the most common species found worldwide, while *S. hircicanis* was reported less often, coinciding with what was reported in goat muscles from China, Egypt, and India (Dafedar et al. 2008; Hu et al. 2016; El-Morsey & Abdo 2024). It is possible to assume that the high infection rates of *S. capracanis* observed worldwide confirmed frequent predation of canids over goats and, in turn, goats frequently ingest water or feed contaminated with canid feces. Despite *S. hircicanis* also using canids as DH, the worldwide prevalence, as well as the positivity rate detected in Argentina, is relatively low (Dafedar et al. 2008; Hu et al. 2016). Potentially, the canids acting as efficient DH are different and predating over goats with less frequency. Suarez et al. (2017) conducted research in northwestern Argentina (NOA), a region that comprises 36% of the country’s goat population, producers were surveyed, revealing that 96.2% owned dogs. We attributed the high *S. capracanis* positivity in the present study to farm management practices, as dogs are commonly utilized on goat farms, serving either as protection against potential threats or as herding dogs for livestock movement. In turn, in these systems, offal and leftovers are consumed by dogs, although it is also likely that they have access to the carcasses or tissues of goats. In goats from Argentina, cystic hydatidosis caused by *Echinococcus granulosus,* transmitted by domestic dogs is the most frequently identified (Cucher et al. 2016), highlighting a frequent goat-dog or prey-predator relationship. All these facts could favor environmental contamination with oocysts/sporocysts from canids (particularly dogs) feces and, therefore, the increased horizontal transmission by the ingestion of oocysts/sporocysts by goats. It could be suggested that wild canids are less frequently predating over goats (especially where dogs are present), and they could be a more efficient DH for *S. hircicanis,* explaining the differences in prevalences and positivities.

Currently, there are no documented reports of *Sarcocystis* spp. infection in goats in Argentina. This study represents the first investigation of *S. capracanis* and *S. hircicanis* in the country, utilizing microscopical and molecular analyses, specifically examining the *18S rRNA* and *coxI* genes of tissue cysts from domestic goats (*Capra aegagrus hircus*). Additionally, this study represents the proof of principle of a serological method utility to evaluate the *Sarcocystis* spp. infection dynamics in goats. Furthermore, further studies to evaluate the potential impact of sarcocystosis on goat production rates in Argentina are underway.

## Conclusion

Based on microscopical and molecular methods, the presence of *S. capracanis* and *S. hircicanis* was confirmed in adult goat muscles in Argentina for the first time. *Sarcocystis* spp. infection is rare in young weaning goats and appears to begin after 8 months. Further research on *Sarcocystis* spp. affecting small ruminants’ validity, their genetic differences, and relative specificity for different canids as DH is needed. Considering the high infection rates and the role of farm dogs in the parasite’s cycle, targeted surveillance and prevention strategies—such as controlling dog access to carcasses and monitoring both goat and dog populations—are recommended to reduce environmental contamination and transmission.

## Supplementary Information

Below is the link to the electronic supplementary material.

ESM 1 (DOCX 9.79 MB)

## Data Availability

The sequences obtained in the frame of this study are reported and available at GenBank (accession numbers: PQ288512, PQ288429, PQ304261, PQ288514, PQ288513, PQ685710, PQ685711, PQ685712, PQ683640, PQ683641, PQ683642, PQ683643, PQ683644, PQ683645).
