# Supplementary material for: Identifying Sarcocystis spp. infection in goats: a combined morphological, serological, and molecular approach
Source: Parasitol Res. 2025 Jun 27;124(6):71. doi: 10.1007/s00436-025-08519-8 (PMC12204907; doi:10.1007/s00436-025-08519-8)
Supplement: Supplementary file 1 — (DOCX 9.79 MB) [file 436_2025_8519_MOESM1_ESM.docx]

**Identifying *Sarcocystis* spp. infection in goats: a combined morphological, serological and molecular approach**

Steffen, K.D.^1, 2, 3^; Gos, M.L.^1, 2^; Helman E.^1, 2^; Unzaga, J.M.^1^; Arias, R.O.^3^; Moré, G.^1, 2, 4*^

1. Immunoparasitology Laboratory, Faculty of Veterinary Science-La Plata National University, La Plata, Buenos Aires (1900), Argentina.

2. National Scientific and Technical Research Council (CONICET), Buenos Aires (1900), Argentina.

3. Animal production department, Faculty of Agrarian and Forest Sciences-La Plata National University, La Plata, Buenos Aires (1900), Argentina.

4. Institute of Parasitology, Vetsuisse Faculty, University of Bern, Länggassstrasse 122, Bern (3012), Switzerland.

*Corresponding Author: [gaston.more@unibe.ch](mailto:gaston.more@unibe.ch)

**Table of contents:**

| Figures | Page Number |
| --- | --- |
| Figure 1 | 2 |
| Figure 2 | 3 |
| Figure 3 | 4 |
| Figure 4 | 5 |
| Figure 5 | 6 |
| Figure 6 | **7** |
| Figure 7 | 8 |
| Tables |  |
| Table 1 | 9 |
| Table 2 | 10-11 |
| Table 3 | 12-13 |

**Figure 1**

**Figure 1.** Goat sampling areas in provinces of Argentina: Buenos Aires (n=79): includes the localities of La Plata (E1, n=69), Villanueva (E2, n=6), and Ranchos (E3, n=4); Salta (n=11): includes the localities of Seclantás (E4, n=7) and Molinos (E5, n=4).

**Figure 2**

**Figure 2.** A) Cyst of *Sarcocystis capracanis* from goat muscle homogenate. Appears thick-walled (<3 μm; opposite white arrows) with radial striations (400x). B) Cyst of *Sarcocystis hircicanis* from goat muscle homogenate. Appears thin-walled (<1 μm; opposite black arrows) with long, filamentous protrusions (hair-like; red arrows) (400x). Scale bar = 50 μm.

**Figure 3**

**Figure 3.** TEM of two longitudinally cut (A, B) from sarcocysts of *Sarcocystis capracanis* in a goat. A) Longitudinal section of the wall of an *S. capracanis* cyst, present in goat muscle (TEM, 15000x): bracket marks the cyst wall (3-5 μm thick) and the palisade-like protrusions (arrows, 3-5 μm long and 0.5-1 μm wide) and a thin ground substance layer (gs, 0.4-0.6 μm). B) Inside view of a sarcocyst. Note an early-formed bradyzoite (Br) with a conoid, nucleus (N), amylopectin granules (Am), rhoptries (Rh), dense granules (Dg), and numerous micronemes (Mic), which appear less dense to the eye, i.e., more electro lucent. Scale bar = 0.5 μm.

**Figure 4**

**Figure 4.** Histopathology of one longitudinally cut of a goat muscle portion showing sarcocysts of *Sarcocystis* spp. A) Histological section of three sarcocysts in goat masseter skeletal muscle; H&E stain (200x), note a sarcocysts (c), connective tissue (ct), and skeletal muscle fiber (f). B) Longitudinal cut of sarcocysts in muscle fiber, note the thin cyst wall (between arrows) (400x). Scale bar = 50 μm.

**Figure 5**

**Figure 5.** Detection of antibodies to *Sarcocystis* spp. by indirect immunofluorescence antibody test (IFAT). The microphotograph shows positive IFAT (presence of antibodies in serum) with complete peripheral green fluorescence of *Sarcocystis cruzi* bradyzoites (visualized as banana-shape, approximately 11 μm long x 4 μm wide, with both ends rounded) white arrows (200x). Scale bar = 50 μm.

**Figure 6**

**Figure 6.** Phylogenetic tree based on *18S rRNA* sequences using the neighbor-joining method. The representative sequences obtained from goats from Argentina are in bold. Phylogenetic analysis based on a multialignment of 35 *18S rRNA* sequences (including four sequences that were used as representatives for the results obtained in the present study) comprising 915bp, placed all *S. capracanis-like* sequences (6003C, PQ685710; 61F, PQ288429; and 4S, PQ288513) in the same branch together with *S. capracanis* and *S. tenella* sequences, while the sequences 4S and 6003C were positioned in a subclade together with *S. capracanis* sequences (Figure 6). The single *S. hircicanis* sequence (61C, PQ288512) was placed in a clade together with *S. hircicanis* and *S. arieticanis* sequences.

**Figure 7**

**Figure 7.** Phylogenetic tree based on *coxI* sequences using the neighbor-joining method. The reference sequences (4S, 5S, and 6003C) obtained from goats from Argentina are in bold. The phylogenetic analysis was based in a multialignment of 30 *coxI* sequences (including 3 of the obtained sequences from goats from Argentina) comprising 1065 bp. The sequences from Argentina (4S, PQ683641; 5S, PQ683642; and 6003C PQ683640) were placed in a clade together with *S. capracanis* sequences with a high support (100%) and as sister clade were positioned the sequences of *S. tenella*. The sequences 4S and 5S are positioned together in a subclade, separated from the sequence 6003C.

**Table 1.** Number and distribution of goats according to location of the establishment, category, sex, and age.

|  |  |  | Sex | | Age |  |  |
| --- | --- | --- | --- | --- | --- | --- | --- |
| Farm | Province | Category | Female | Male | years/days | No. of samples | Total |
| E1 | Buenos Aires | Adults | 5 | - | ≥4 | 5 | 19 |
| E2 |  |  | 3 | - | ≥3 | 3 |  |
| E3 |  |  | 1 | - | 1.5 | 1 |  |
| E4 | Salta |  | 6 | - | ≥1.5 | 6 |  |
| E5 |  |  | 4 | - | ≥4 | 4 |  |
| E1 | Buenos Aires | Kids | 9 | 54 | f=67 to 290; m=37 to 188 | 63 | 71 |
| E2 |  |  | 1 | 2 | f=94; m=74 to 83 | 3 |  |
| E3 |  |  | 1 | 2 | f=90; m=90 to 97 | 3 |  |
| E4 | Salta |  | - | 1 | m=96 | 1 |  |
| E5 |  |  | - | 1 | m=120 | 1 |  |
| Total |  |  | 30 | 60 |  |  | 90 |

References: f= female, m= male

**Table 2.** *Sarcocystis* spp. IFAT titers and the presence of microscopic cysts in the muscle of goats according to establishment, age, and sex.

|  |  |  |  | IFAT titers | Light microscopy |
| --- | --- | --- | --- | --- | --- |
| Nº-ID | Farm | Goats Age | Sex | *Sarcocystis* spp. | Microscopic cysts |
| 1-61 | E1 | >6 | F | Negative | Yes |
| 2-63 | E1 | >6 | F | Negative | Yes |
| 3-68 | E1 | >6 | F | 100 | Yes |
| 4-288 | E1 | >4 | F | 100 | Yes |
| 5-5004 | E2 | >6 | F | 100 | Yes |
| 6-8072 | E2 | >3 | F | 100 | Yes |
| 7-cap22 | E3 | 1.5 | F | 100 | Yes |
| 8-MATC1 | E4 | >3 | F | Negative | Yes |
| 9-MATC2 | E4 | 1.5 | M | 50 | Yes |
| 10-MATC3 | E4 | 1.5 | M | 50 | Yes |
| 11-MATC4 | E4 | 1.5 | M | 100 | Yes |
| 12-MATC5 | E4 | 1.5 | M | 100 | Yes |
| 13-MATC6 | E4 | <1 | M | Negative | No |
| 14-MATC7 | E4 | >2 | F | 50 | Yes |
| 15-EAC1 | E5 | >5 | F | Negative | Yes |
| 16-EAC2 | E5 | <1 | M | Negative | No |
| 17-EAC3 | E5 | >5 | F | 100 | Yes |
| 18-EAC4 | E5 | >4 | F | 100 | Yes |

Reference: E1-5= Establishments 1 to 5; IFAT: Immunofluorescence indirect antibody test.

**Table 3**. Sequencing of fragments from *18S rRNA* and *coxI* genes results from goat's individual sarcocyst samples.

| **Sample ID** | **Province and Farm** | ***18S rRNA***  **BLAST**  **identity** | ***coxI***  **BLAST**  **identity** | **Sequence**  **length (Bp)**  ***18S/coxI*** | **GenBank**  **Accession number *18S/coxI*** |
| --- | --- | --- | --- | --- | --- |
| 61 C* | BA  E1 | 100% *S. hircicanis* (KU820984) | - | 818/- | PQ288512*/ - |
| 61 F* | BA  E1 | 99.76% *S. capracanis* (L76472; MW832485) | 98.89% *S. capracanis* (MW848331) | 843/542 | PQ288429*/PQ683645 |
| 68 1C | BA  E1 | 99.76% *S. capracanis* (L76472; MW832485) | - | 818/- | - |
| 5004 1A | BA  E2 | 99.88% *S. capracanis* (KU820982; MW832493) | - | 841/- | - |
| 8072 D | BA  E2 | 99.88% *S. capracanis* (MW832491; PQ963169) | - | 829/- | PQ304261/ - |
| 6003 C* | BA  E2 | 100% *S. capracanis* (MW832494; MW832480) | 99.41% *S. capracanis* (MW848328) | 838/1020 | PQ685710*/PQ683640* |
| 6003 F | BA  E2 | 100% *S. capracanis* (MW832478; PQ963169) | - | 736/- | - |
| 6003 K | BA  E2 | 100% *S. capracanis* (MW832494; MW832480) | - | 576/- | - |
| 2 S | ST  E4 | 100% *S. capracanis* (MW832478; PQ963169) | 99.13% *S. capracanis* (MW848328) | 620/1038 | PQ288514/PQ683643 |
| 3 S | ST  E4 | 100% *S. capracanis* (MW832486; PQ963169) | - | 614/- | - |
| MATC4/R | ST  E4 | 100% *S. capracanis* ([L76472](https://www.ncbi.nlm.nih.gov/nucleotide/L76472.1?report=genbank&log$=nucltop&blast_rank=1&RID=BX0GNEBV013)) | 99.81% *S. capracanis* (MW848328) | 586/1038 | PQ685711/PQ683644 |
| MATC5/F | ST  E4 | 99.81% *S. capracanis* ([MW832494](https://www.ncbi.nlm.nih.gov/nucleotide/MW832494.1?report=genbank&log$=nucltop&blast_rank=3&RID=BXW590JT013); KR155199) | - | 517/- | - |
| 4 S* | ST  E5 | 99.64% *S. capracanis* (MW832483) | 99.71% *S. capracanis* (MW848328) | 845/1035 | PQ288513*/PQ683641* |
| 5 S* | ST  E5 | 99.70% *S. capracanis* (MW832493; MW832487) | 99.81% *S. capracanis* (MW848328) | 664/1038 | PQ685712/PQ683642* |
| 6 S | ST  E5 | 100% *S. capracanis* (MW832494, MW832480) | - | 546/- | - |

References: Province: BA: Buenos Aires, ST: Salta; E1-5= Establishments 1 to 5; Bp= Sequence base pairs; *: Samples and GenBank accession numbers marked with an asterisk were used as a reference for constructing the phylogenetic trees.
